# Recurrent Migratory Transient Bone Marrow Edema of the Knees Associated with Low Vitamin D and Systemic Low Bone Mineral Density: A Case Report and Literature Review

**DOI:** 10.1155/2018/7657982

**Published:** 2018-02-18

**Authors:** Omar Alsaed, Mohammad Hammoudeh

**Affiliations:** Hamad Medical Corporation, Hamad General Hospital, Rheumatology Section, P.O. Box 3050, Doha, Qatar

## Abstract

Transient bone marrow edema (TBME) is a self-limiting disease characterized by joint pain with localized bone marrow edema by MRI and has been reported in many case series and case reports. It is well known that joints of the lower extremity including hips, knees, ankles, and feet are the classical sites for TBME. Many theories have been proposed for the pathogenesis of TBME. Systemic osteopenia and vitamin D deficiency is one of the theories that have been suggested in the last few years. In this case report, we present a middle-aged male patient, who presented with 4 attacks of TBME in both knees between September 2016 and August 2017. The patient was found to have persistently low vitamin D and osteopenic *T* score in DXA scan of the lumbar spine and hips. Patients of TBME usually present with joint pain that is provoked by weight-bearing physical activity. The aim of this case report is to raise the awareness that TBME can be the initial presentation of systemic loss of bone mineral density.

## 1. Introduction

Bone marrow edema (BME) is a magnetic resonance imaging (MRI) descriptive terminology. It is a nonspecific finding that can be caused by many conditions that affect the joint and the periarticular bone [1]. Inflammatory arthritis, infections, malignancies, and fractures are common causes of BME. Usually BME of these conditions resolves after treating the underlying cause. In certain conditions, BME can present as a self-limited joint pain that resolves over months without any sequelae and without identifying any underlying causes. In such cases, it is called transient BME (TBME) and is commonly reported in the hips; however, knees, ankles, and feet can be involved as well [2–6]. Recurrence of the TBME has been described in some reports; it may occur in the primary joint, or it may spread to another joint as well. Interestingly, involvement of another compartment of the primary joint can also occur [7], similar to our case. In such a scenario, TBME is called migratory transient bone marrow edema. The recurrence and the migratory nature of this disease raise the suspicion of the underlying systemic cause. The link between TBME and systemic loss of bone mineral density and vitamin D deficiency has been described in few cases [8, 9]. Localized osteoporosis at the site of bone marrow edema was confirmed by dual-energy X-ray absorptiometry (DXA) scan in many case reports [10]. Although DXA shows osteoporosis in some cases, it is not clear if the systemic low bone mineral density is a risk factor for the development of microfracture and bone marrow edema. On the other hand, microfracture might itself cause the edema and separation of the bone trabeculae, reported as localized osteoporosis on DXA scan.

The aim of reporting this case is to show the association between TBME and systemic bone mineral density loss and also to raise the awareness of the importance of osteopenia/osteoporosis screening in patients presented with TBME.

## 2. Case Presentation

A 47-year-old male patient was referred to the rheumatology clinic because of recurrent attacks of pain in both knees over 1 year.

**Figure 1:**
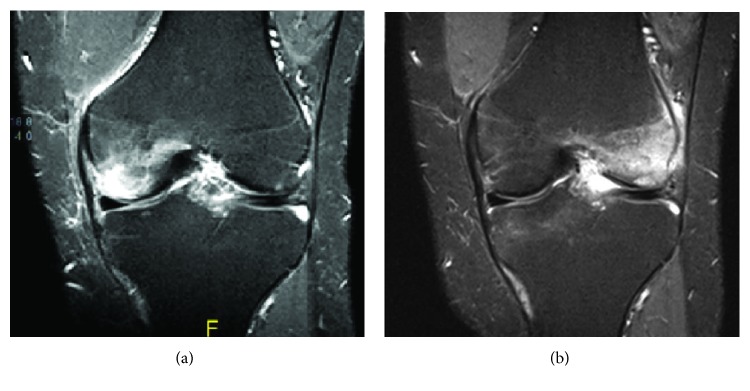
MRI of the left knee (a) in September 2016 and (b) three months later.

In September 2016, the patient presented with severe pain over the medial aspect of the left knee for a two-week duration which prevented him from ambulation. The pain increased with weight-bearing physical activity. The patient reported no history of trauma before the onset of the knee pain. Examination showed severe tenderness over the medial side of the knee with mild effusion and moderate limitation of range of motion. There was no erythema or increased warmth of the knee. MRI of the left knee showed a moderate-sized focal area of marrow edema/contusion involving the medial femoral condyle in mid and anterior parts predominantly along the articular surface. The patient was prescribed diclofenac sodium 50 mg twice daily and was advised to avoid prolonged weight-bearing activities. Over the next few weeks, the pain subsided and resolved. Three months later, the patient developed spontaneous new onset of pain involving the lateral aspect of the same knee. MRI showed bone marrow edema involving the lateral femoral condyle with complete resolution of the bone marrow edema of the medial femoral condyle. He was treated conservatively with NSAIDs and physiotherapy and advised to use cane to minimize weight bearing on the diseased knee.  Figure 1 demonstrates MRI of the left knee in September 2016 and three months later.

**Figure 2:**
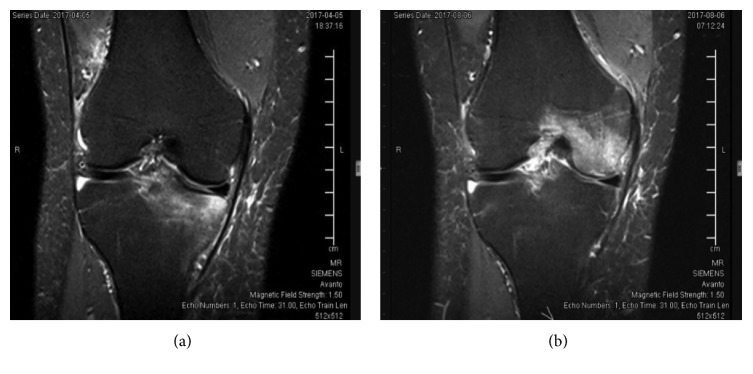
MRI of the left knee (a) in April 2017 and (b) four months later.

In April 2017, the patient developed gradual pain over the medial side of the right knee with no obvious swelling. MRI of the right knee showed a moderate-sized focal area of marrow edema involving the medial tibial plateau medially and anteriorly. The patient was treated conservatively in a similar fashion to the previous episode. Four months later, the pain got more severe for which he underwent another MRI of the right knee which showed extensive marrow edema involving the medial femoral condyle with complete recovery of the medial tibial plateau bone marrow edema noted in the previous MRI (Figure 2). The patient also recalled a similar pain happened in 2011 to the left knee but did not do MRI at that time.

In all previous presentations, the patient did not report any history of trauma, fall, twist, constitutional symptoms, or using corticosteroids. He also had no history of other joint involvement apart from knees and denied any history of low back pain. He did not have any features suggestive of spondyloarthropathy or connective tissue disease.

Past history is significant for fracture of the greater tuberosity of the left humerus and undisplaced fracture of the left cuboid bone. Fractures happened after he fell off a ladder. Also, he is known to have mild asthma which is controlled with as-needed bronchodilator and hypertension maintained on amlodipine 5 mg daily. The patient had never been a smoker or an alcohol consumer.

Lab investigations revealed vitamin D 8 ng/mL (normal: >30 ng/mL), corrected calcium 2.16 mmol/L (normal: 2.10–2.60 mmol/L), parathyroid hormone 91 pg/ml (normal: 15–65 pg/ml), and alkaline phosphatase 49 U/L (normal: 40–150 U/L). Complete blood count, kidney and liver function, CRP, and ESR were within normal limit. Immunology profile including rheumatoid factor, ACPA, ANA, anticardiolipin, and B_2_-glycoprotein were all negative.

DXA scan showed a *T* score of −1.0 at the lumbar spine and −1.6 at the left femoral neck suggestive of osteopenia.  Table 1 shows further details of the DXA scan.

The patient was treated conservatively with oral vitamin D2 50,000 IU/week supplement and NSAIDs. Gradually, the symptoms subsided over the next few weeks, and vitamin D level became normal after 12 weeks.

## 3. Discussion

We presented a case of a 47-year-old male patient who presented with 4 recurrent attacks of TBME in both knees within one-year period associated with vitamin D deficiency and systemic low bone mineral density.

The number of published cases of TBME of the knee that underwent DXA scan screening for systemic osteoporosis is limited. In 2002, Trevisan et al. [3] published 3 cases with TBME at different sites (hip, knee, and ankle). All of them are male at fourth and fifth decades. Severe osteoporosis at the hip and lumbar spine was found in all of them. Ringe et al. [4] studied 12 patients with TBME who underwent lumbar and femoral neck DXA scan; 5 had osteopenia, 3 had osteoporosis, and 4 were normal. The majority of the patients in this case series had TBME in the hip and ankle. Sprinchorn et al. [5] in 2011 reported ten patients with TBME in the ankles. Four patients were found to have osteoporosis and five had osteopenia. Only one patient had normal bone density. Serum vitamin D levels were low in nine patients and normal in one patient. Eight patients that developed osteopenia or osteoporosis were female at the age of postmenopause and above. In a more recent study on TBME of the hip done by Klontzas et al. [6], 31 patients had lumbar densitometry evaluation resulting in 15 patients being classified as osteopenic and 15 as osteoporotic. In 2016, again Trevisan et al. [9] published 23 cases of TBME (13 hips, 6 knees, 2 ankles, and 2 feet). Ten out of the 23 (5 males and 5 females) underwent DXA scan for hips and the lumbar spine. Six patients (4 TBME hips and 2 knees) out of the ten were found to have osteopenia or osteoporosis. Abstract of few case reports published in 1986 [11] and 1990 [12] showed TBME with systemic osteoporosis. Full manuscript of these case reports is not available.

Many theories have been proposed for the underlying cause of TBME. Ischemic insult is hypothesized as an underlying cause for an early or mild form of osteonecrosis presented as TBME. This was supported by many histological studies of TBME at the hip joint [13]. However, in one study of 155 patients diagnosed with TBME of the hip by imaging criteria, none of the patients progressed to osteonecrosis, casting doubt on the suggestion that TBME is part of a clinical spectrum that may result in either spontaneous resolution (TBME) or avascular bone death [6]. Another point which does not support this hypothesis is that patients who are at high risk for ischemic bone necrosis never presented with TBME. For example, sickle cell disease usually presents with bone necrosis rather than TBME.

Due to histological similarities between TBME and complex regional pain syndrome (CRPS), some authors believe that TBME is a nontraumatic form of CRPS [14]; however, differences in clinical and radiological findings disapprove this hypothesis. For instance, patients with CRPS usually present with vasomotor and neurological symptoms in addition to pain and soft tissue swelling. History of trauma is an important clinical key for CRPS diagnosis which is not the case for TBME.

The association of TBME and reduced systemic bone mineral density is reported in few of the cases. This led some investigators to formulate a theory suggesting that osteopenia and osteoporosis are the underlying etiologies of TBME. But due to the quality and the quantity of the available evidence, a conclusion cannot be reached. It needs large epidemiological studies or case-controlled studies.

The prevalence of TBME is underestimated due to many reasons: the nonspecificity of clinical presentation, the short duration of the illness, good prognosis with over-the-counter pain killer, and limited access to MRI, all lead to underestimate the prevalence and less case identification to be enrolled in the controlled study.

Our patient was found to have severe vitamin D deficiency, elevated PTH, and low normal calcium as he was not compliant with vitamin D supplement. The link between vitamin D deficiency and TBME is debatable. As we mentioned earlier, Sprinchorn [5] found that 90% (9/10) of the TBME cases had low vitamin D. In a larger retrospective study done by Horas et al. [8], in 2017, 84% of patients (26/31) had low vitamin D levels. Some authors question the link between low vitamin D and TBME for two reasons: first, due to high prevalence rate of vitamin D deficiency in general population which was proven in many studies. The overall prevalence rate of vitamin D deficiency was 41.6% in a big study done in 2011 in US population [15]. The rate was even higher in the Middle East. It is around 80% in Saudi Arabia [16]. Second, TBME is a rare disease, and the prevalence is underestimated for the reasons that were mentioned earlier.

In conclusion, low bone mineral density is commonly reported in transient bone marrow edema, which makes screening and treatment of osteopenia and osteoporosis to be recommended.

In view of the previous data and discussion, large epidemiological studies are needed to confirm that vitamin D deficiency and systemic loss of bone mineral density could be a risk factor for TBME.

## Figures and Tables

**Table 1:** DXA scan of the lumbar spine and left hip.

Region	BMD (g/cm^2^)	*T* score	*Z* score
Lumbar spine	0.980	−1.0	−0.7
Left neck of the femur	0.710	−1.6	−0.9
